# Combining Immunocytokine and *Ex Vivo* Activated NK Cells as a Platform for Enhancing Graft-Versus-Tumor Effects Against GD2^+^ Murine Neuroblastoma

**DOI:** 10.3389/fimmu.2021.668307

**Published:** 2021-08-19

**Authors:** Paul D. Bates, Alexander L. Rakhmilevich, Monica M. Cho, Myriam N. Bouchlaka, Seema L. Rao, Joanna M. Hales, Rimas J. Orentas, Terry J. Fry, Stephen D. Gilles, Paul M. Sondel, Christian M. Capitini

**Affiliations:** ^1^Department of Pediatrics, University of Wisconsin School of Medicine and Public Health, Madison, WI, United States; ^2^Department of Human Oncology, University of Wisconsin School of Medicine and Public Health, Madison, WI, United States; ^3^Pediatric Oncology Branch, Center for Cancer Research, National Cancer Institute, National Institutes of Health, Bethesda, MD, United States; ^4^Department of Pediatrics, University of Washington School of Medicine, Seattle, WA, United States; ^5^Ben Towne Center for Childhood Cancer Research, Seattle Children’s Research Institute, Seattle, WA, United States; ^6^Department of Pediatrics, University of Colorado School of Medicine, Aurora, CO, United States; ^7^Provenance Biopharmaceuticals, Carlisle, MA, United States; ^8^Carbone Comprehensive Cancer Center, University of Wisconsin School of Medicine and Public Health, Madison, WI, United States

**Keywords:** immunocytokine, NK cells, neuroblastoma, graft-versus-tumor effect, cytokine release syndrome

## Abstract

Management for high-risk neuroblastoma (NBL) has included autologous hematopoietic stem cell transplant (HSCT) and anti-GD2 immunotherapy, but survival remains around 50%. The aim of this study was to determine if allogeneic HSCT could serve as a platform for inducing a graft-versus-tumor (GVT) effect against NBL with combination immunocytokine and NK cells in a murine model. Lethally irradiated C57BL/6 (B6) x A/J recipients were transplanted with B6 bone marrow on Day +0. On day +10, allogeneic HSCT recipients were challenged with NXS2, a GD2^+^ NBL. On days +14-16, mice were treated with the anti-GD2 immunocytokine hu14.18-IL2. In select groups, hu14.18-IL2 was combined with infusions of B6 NK cells activated with IL-15/IL-15Rα and CD137L *ex vivo*. Allogeneic HSCT alone was insufficient to control NXS2 tumor growth, but the addition of hu14.18-IL2 controlled tumor growth and improved survival. Adoptive transfer of *ex vivo* CD137L/IL-15/IL-15Rα activated NK cells with or without hu14.18-IL2 exacerbated lethality. CD137L/IL-15/IL-15Rα activated NK cells showed enhanced cytotoxicity and produced high levels of TNF-α *in vitro*, but induced cytokine release syndrome (CRS) *in vivo*. Infusing Perforin^-/-^ CD137L/IL-15/IL-15Rα activated NK cells had no impact on GVT, whereas TNF-α^-/-^ CD137L/IL-15/IL-15Rα activated NK cells improved GVT by decreasing peripheral effector cell subsets while preserving tumor-infiltrating lymphocytes. Depletion of Ly49H^+^ NK cells also improved GVT. Using allogeneic HSCT for NBL is a viable platform for immunocytokines and *ex vivo* activated NK cell infusions, but must be balanced with induction of CRS. Regulation of TNFα or activating NK subsets may be needed to improve GVT effects.

## Introduction

Neuroblastoma (NBL) is the most common extracranial solid tumor that occurs in children. For children with tumors that either have high risk biologic features or with metastatic disease, overall survival is still poor despite an aggressive treatment regimen that includes chemotherapy, surgery, autologous hematopoietic stem cell transplant (HSCT), radiation, and maintenance therapy with cis-retinoic acid (1). The addition of the monoclonal antibody (moAb) dinutuximab (which targets the NBL-associated antigen GD2), interleukin-2 (IL-2) and granulocyte-monocyte colony stimulating factor (GM-CSF) improved event-free and overall survival (2), but is not curative for the majority of patients who will ultimately relapse and die. Another treatment approach is needed that can potentially improve survival further and lead to a long-term cure.

Allogeneic HSCT was initially used in children with NBL about 30 years ago with some reports of clinical responses (3), but was never shown superior to autologous HSCT (4–6). Because there has not been convincing evidence of a graft-versus-tumor (GVT) effect against NBL (6–8), and because allogeneic HSCT introduces the life-threatening potential for graft-versus-host-disease (GVHD), autologous HSCT remains the standard of care. In fact, current protocols are incorporating tandem autologous HSCTs as consolidative therapy to improve event-free survival (9). Because of both preclinical evidence (10, 11) and case reports suggesting some clinical benefit of allogeneic HSCT in NBL, particularly in the haploidentical setting (12, 13), the objective of this preclinical study was to incorporate haploidentical HSCT as a platform for a combined immunotherapy regimen to enhance the GVT effect against NBL.

Until 2019, dinutuximab was given with GM-CSF and IL-2 in the Children’s Oncology Group (COG) as separate treatments as part of upfront maintenance therapy for NBL. Due to excessive toxicity associated with systemic IL-2 administration without clear added benefit, COG eliminated usage of IL-2 and now dinutuximab and GM-CSF are used. One means by which to maintain the beneficial activation of IL-2 for antibody-dependent cellular cytotoxicity (ADCC) without systemic toxicity is to restrict its use to the immune synapse. Hu14.18-IL2 is a fusion protein consisting of human IL-2 genetically linked to the carboxyl-termini of each human IgG1 heavy chain of the GD2-specific hu14.18 moAb. This “immunocytokine” (IC) provides a local source of IL-2 at the immunological synapse between the effector cell and the NBL, activating immunity against GD2^+^ tumors. Hu14.18-IL2 has been used in both phase I and phase II trials in children with refractory NBL and melanoma, with reversible toxicities and complete responses observed in both phase II NBL trials (14, 15). However, hu14.18-IL2 therapy is not curative when used as a single agent to treat macroscopic refractory or recurrent NBL, and has never been tested after allogeneic HSCT. The mechanism of action for hu14.18-IL2 is thought to be, at least in part, from ADCC from natural killer (NK) cells (16).

Because of the availability of clinical grade cytokines and artificial antigen presenting cells (aAPCs), infusion of high numbers of purified, *ex vivo* activated NK cells are emerging from preclinical models into clinical trials. NK cells have already been shown to have cytotoxicity *in vitro* against a variety of NBL cell lines (17) and primary patient tumors (18, 19) as well as *in vivo* with xenograft NBL models (20). In addition, the lymphopenic environment induced from the conditioning regimen for allogeneic HSCT is conducive for NK cell expansion given the presence of high levels of IL-15 (21). Lastly, NK cells produce growth factors like IL-1β, IL-6, G-CSF and GM-CSF that can support engraftment (22).

NK cells possess inhibitory receptors on their cell surface that can “turn off” the cells when they engage major histocompatibility complex (MHC) antigens (23, 24). Our current standard of administering an anti-GD2 moAb (dinutuximab) after autologous HSCT is limited in that the patient’s own NK cells must engage the antibody to eliminate the tumor, and risk engaging self-MHC on the tumor that could “turn off” the NK cell. In fact, two studies in children with NBL who were treated with anti-GD2 based therapies (one with hu14.18-IL2 and one with the moAb 3F8) reported a better response to therapy in those patients that were self-killer immunoglobulin-like receptor (KIR)/KIR ligand mismatched (25, 26), something that can be easily achieved if NK cells came from an appropriately selected allogeneic donor. In this study, we explore haploidentical allogeneic HSCT in NBL-bearing mice as a means of insuring that some of the inhibitory Ly49 receptors on donor murine NK cells do not engage their cognate MHC ligand, potentially “turning on” the NK cells and maximizing anti-tumor activity after hu14.18-IL2 IC administration.

## Materials and Methods

### Mice

C57BL/6NCr (B6, H-2^b^), Balb/cAnNCr (Balb/c, H-2^d^), CB6F1/Cr (CB6F1, H-2^b x d^), B6Ly5.2/Cr (CD45.1^+^ B6, H-2^b^), A/JCr (A/J, H-2^a^), and DBA/2NCr (DBA, H-2^d^) mice were purchased from the National Cancer Institute (NCI) Animal Production Program and Charles River Laboratories International (Frederick, MD). B6AJF1 (H-2^b x a^), C57BL/6-Prf1<tm1Sdz>/J (Perforin^-/-^, H-2^b^) and B6.129S-Tnf<tm1Gkl>/J (TNFα^-/-^, H-2^b^) were purchased from the Jackson Laboratory (Bar Harbor, ME). Mice were female and used between 8 and 16 weeks of age. All animals were housed in a pathogen-free facility throughout the study. The Animal Care and Use Committees (ACUC) at the University of Wisconsin (M005915, M01246) and National Institutes of Health (PB027) approved all protocols.

### Bone Marrow Transplantation (BMT)

On BMT Day +0, bone marrow (BM) cells were harvested from donor mice and T cell depleted as previously described (27). BM recipients were lethally irradiated with a single fraction of 1100 rads (B6AJF1) or 1000 rads, (B6), 800 rads divided in two 400 rad fractions separated 4 hours apart (Balb/c) or 750 rads divided into two 375 rad fractions separated 4 hours apart (A/J). Irradiated BM recipients were then injected intravenously (i.v.) with 5 x 10^6^ CD3-depleted BM cells in serum-free RPMI (Invitrogen, Carlsbad, CA). In select groups, T cells from donor mice were isolated from spleens using magnetic cell selection (Miltenyi Biotec, Auburn, CA), and injected i.v. with the BM. Mice were weighed individually biweekly, and the mean weight of each treatment group was calculated at each time point and compared with the day +0 weight. GVHD was monitored using a clinical scoring system (28). Veterinarians and veterinary technicians who were blinded to the experimental groups examined for moribund mice, and assessed the mice daily in accordance with approved institutional protocols.

### Tumor Cell Lines

NXS2 is a murine GD2^+^ NBL cell line on an H-2^a^ background (29), and was obtained from Ralph Reisfeld (Scripps Research Institute). N18TG2 is also murine GD2^+^ NBL cell line on an H-2^a^ background and was obtained from Sigma-Aldrich, Inc (St. Louis, MO). Neuro-2a is a murine GD2^-^ NBL cell line on an H-2^a^ background, and was obtained from ATCC (Manassas, VA). 9464D is a murine GD2^+^ NBL cell line on an H-2^b^ background, and was obtained from Jon Wigginton (while previously at the National Cancer Institute, Frederick, MD). Yac-1 is a murine B cell lymphoma cell line on an H-2^a^ background and was obtained from ATCC. A20 is a murine B cell lymphoma cell line on an H-2^d^ background and was obtained from ATCC. Cell authentication was performed using short tandem repeat analysis (Idexx BioAnalytics, Westbrook, ME) and per ATCC guidelines using morphology, growth curves, and *Mycoplasma* testing within 6 months of use using the e-Myco mycoplasma PCR detection kit (iNtRON Biotechnology Inc, Boca Raton, FL). All tumor cells were maintained in culture at 37°C in 5% CO_2_ in Complete Mouse Media (CMM), and used after 3-5 passages in culture after thawing.

### *In Vivo* Tumor Challenge

Exponentially growing NBL tumor cells were prepared as a single cell suspension in serum-free RPMI and injected into the subcutaneous fat of the shaved flank at a dose of 2 × 10^6^ tumor cells on day +10 after HSCT. Tumors were measured in 2 dimensions (length × width) 1-2 times a week by digital caliper. Tumor growth = length x width (mm^2^). Mice were euthanized with CO_2_ when tumor diameters reached 2 cm in any dimension, in accordance with animal protocols. If a mouse was found dead, the previously recorded tumor measurement was carried for the rest of the data points of the experiment for the purposes of statistical comparison. Exponentially growing A20 tumor cells were prepared as a single cell suspension in serum-free RPMI and injected as 2.5 x 10^6^ cells i.v. on Day +5 into Balb/c mice.

### NK Cell Isolation and Activation

NK cells were purified from single cell suspensions of spleens using magnetic cell selection (Miltenyi Biotec) and placed into CMM and 10ng/mL recombinant IL-15/IL15Rα complex (eBioscience, San Diego, CA) at 37°C in 5% CO_2_. Because IL-15 is typically presented in trans by IL-15Rα, the complex was utilized to potently increase IL-15 bioactivity. Artificial antigen presenting cells (aAPCs) consisted of irradiated (10,000 rads) Yac-1 cells or Yac-1 cells transfected with CD137L (Yac1-CD137L) (30). For *in vivo* infusions, NK cells were incubated with aAPCs at a 1:1 ratio for 1 week, then washed in PBS and resuspended in serum-free RPMI prior to injection. IL-15/IL-15Rα was replaced two times per week.

### Adoptive NK Cell Infusions and Immunocytokine Therapy

In select experiments, allogeneic HSCT recipients were treated on day +14 with *ex vivo* IL-15/IL-15Rα or CD137L/IL-15/IL-15Rα activated NK cells. On days 14-16, select groups were also treated with either PBS or 50mcg hu14.18-IL2 i.v. (Apeiron Biologics, Vienna, Austria) alone or in combination with CD137L/IL-15/IL-15Rα NK cells.

### Ly49H^+^ NK Depletion

Ly49H^+^ NK cells were depleted using a purified 3D10 clone (Biolegend Cat # 144704) (31). Basically, B6AJF1 mice were transplanted as above with B6 BM and challenged with NXS2 tumors on Day +10. On Day +12, 48hrs before NK injection, 200ug of anti-Ly49H or IgG1 isotype control was given IP per mouse. On Day +14, 1 x 10^6^ CD137L/IL-15/IL-15Rα NK cells were infused IV per mouse with 50ug hu14.18-IL2, and mice were followed for tumor growth.

### Flow Cytometric Analysis

In brief, 1 × 10^6^ freshly isolated, erythrocyte-depleted splenocytes, lymph node, BM cells, or expanded NK cells were stained at 4°C for 20 minutes with a monoclonal antibody cocktails containing either NK1.1-PerCP Cy5.5 (Cat # 108728) or NK1.1-PE (Cat # 108708) (BioLegend, San Diego, CA), Ly49C/I-FITC (Cat # 553276), Ly49H-FITC (Cat # 562536) (BD-Biosciences, San Jose, CA) or Ly49H-PE-Cy7 (Cat # 144714, BioLegend), B220-BV421 (Cat # 103251, BioLegend), CD4-eFluor 450 (Cat # 48-0048-42, Thermo Fisher Scientific), CD45.2-PerCP Cy5.5 (Cat # 109828, BioLegend) or CD45.2-FITC (Cat # 109806, BioLegend), CD8-PE (Cat # 100708, BioLegend) or CD8-APC (Cat # 100712, BioLegend), CD45.1-APC (Cat # 110714, BioLegend) or CD45.1-Pacific Blue (Cat # 110722, BioLegend), GD2 PE (Cat# 357304, BioLegend), FasL-APC (Cat # 106610, BioLegend), and TRAIL-PerCP-Cy5.5 (Cat # 109314, BioLegend) and then washed in fluorescence-activated cell sorting (FACS) buffer (phosphate-buffered salt solution with 0.2% fetal calf serum and 0.1% sodium azide). For degranulation and intracellular cytokine analysis, expanded NK cells were incubated with or without PMA (50 ng/ml) and ionomycin (1 ug/ml) for 1 hour 37°CC in 5% CO_2_. Then GolgiSTOP (monesin) and GolgiPLUG (brefeldin A) were added and the cells were incubated for an additional 4 hours. Cells were then harvested and stained with surface monoclonal antibodies. This was followed by fixation and permeabilization using the BD Cytofix/Cytoperm Fixation/Permeabilization Kit (Cat # 554714, BD) and staining with monoclonal antibody TNFα-AF647 (Cat # 506314, BioLegend). Flow cytometry data was acquired on a MACSQuant analyzer 10 (Miltenyi Biotec) and mqd files were converted to fcs files using The MACSQuantify™ Software or Attune NxT flow cytometer (Thermo Fisher). Listmode data were analyzed using FlowJo software (FlowJo, Ashland, OR).

### Cytokine Production

For *in vitro* studies, NK cells were expanded with 10ng/mL IL-15/IL-15Rα alone or with Yac1-CD137L and IL-15/IL-15Rα for 1 week, then cultured at 1 x 10^6^ cells/ml in CMM at 37°CC in 5% CO_2_ for 3 hours. Supernatants were harvested and analyzed by enzyme-linked immunosorbent assays (ELISA) for murine TNFα (R&D Systems, Minneapolis, MN) according to manufacturer’s instructions. ELISA plates were read on a VersaMAX Microplate Reader at 450nm and analyzed using SoftMAX Pro 5 reader (Molecular Devices, Sunnyvale, CA). For *in vivo* studies, allogeneic HSCT mice had peripheral blood collected by heel stick. Serum was isolated and frozen at -20°C until used in a V-Plex Plus Pro-Inflammatory Panel 1 mouse kit according to manufacturer’s directions (Meso Scale Diagnostics, Rockville, MD). Samples were run in duplicate on a MesoQuickplex SQ 120 multiplex cytokine analyzer (Meso Scale Diagnostics).

### Cytotoxicity Assays

Cytotoxicity is performed using a Promega CytoTox 96 Non-Radioactive Cytotoxicity assay. Cytotoxic activity is colorimetrically measured by the amount of lactate dehydrogenase (LDH) released by the cells plated within a 96 well plate. Color formed by lysed cells is measured by wavelength absorbance (490nm). Cells plated in the assay are a ratio concentration of effector cells (NK cells) to target cells (tumor cells) diluted 2-fold starting at 20:1 to 5:1 effector:target (E:T) ratio. Effectors and target cells were co-incubated for 4 hours at 37°C before measuring wavelength absorbances on a VersaMAX Microplate Reader. Spontaneous release was determined by adding 100 µl of media to 100 µl of tumor cells. Maximum LDH release was determined by adding 100 µL of 1X-Triton X-100 detergent (Sigma-Aldrich, cat#: 9002-93-1) to tumor cells. Specific LDH release was calculated as: % lysis = 100% x (Experimental–Spontaneous)/(Maximum–Spontaneous). Additional cytotoxicity assays were performed using a calcein-AM release assay. Cytotoxic activity was measured by the amount of calcein released from lysed target cells plated within a 96 well plate. Cells were plated in the assay at a 5:1 E:T ratio. Following co-incubation for 4 hours at 37°C, supernatant calcein signal was measured using a fluorescent plate reader at 495/515 nm. Maximum calcein release was determined by adding 100 µL of 1X-Triton X-100 detergent to tumor cells and % lysis was calculated as above.

### Statistical Analysis

Statistics were performed using GraphPad Prism version 9.0 for the Macintosh OS (GraphPad Software, San Diego, CA). Significant differences when comparing 2 groups were determined by the 2-tailed Mann–Whitney test or unpaired t test with Welch’s correction. The Kruskal–Wallis with Dunn’s multiple-comparison post-test was used to assess statistical differences among 3 or more groups. Survival analysis was plotted according to the Kaplan-Meier method, and statistical differences were determined with the log-rank test. A p value less than 0.05 was considered statistically significant.

## Results

**Figure 1:**
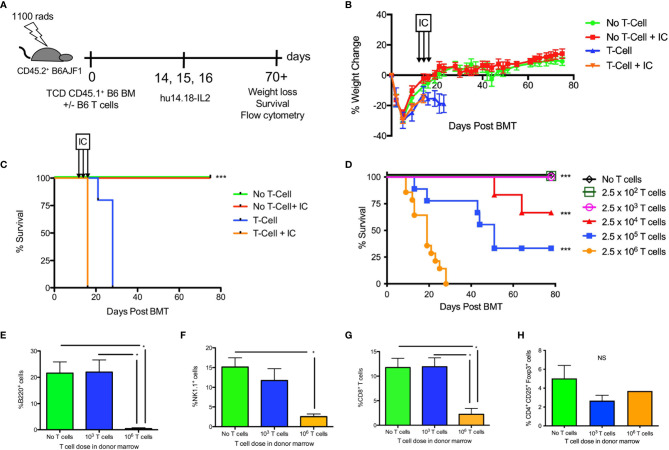
Effect of hu14.18-IL2 after T cell deplete and replete allogeneic HSCT. **(A)** Lethally irradiated CD45.2^+^ B6AJF1 mice (H-2^b x a^) were transplanted with: either CD3e cell depleted (No T cell) or CD3e depleted BM replenished with 2.5 x 10^6^ T cells (T cell) from congenic CD45.1^+^ B6 mice (H-2^b^) on Day +0. On days +14-16, PBS or hu14.18-IL-2 (IC) was administered and allogeneic HSCT recipients were followed for **(B)** GVHD-associated weight loss and **(C)** survival. N=5 mice/group. The no T cell group was compared to the corresponding T cell group. **(D)** Lethally irradiated CD45.2^+^ B6AJF1 mice were transplanted with either no T cells or CD3e depleted BM from congenic CD45.1^+^ B6 mice replenished with logarithmically increasing doses of T cells (2.5 x 10^2^-10^6^) on Day +0. On days +14-16, IC was administered and allogeneic HSCT recipients were followed for survival. Each group was compared to the 2.5 x 10^6^ T cell group. Results pooled from 2 similar experiments, 5-10 mice/group. **(E)** Lethally irradiated CD45.2^+^ B6AJF1 mice were transplanted with either no T cells or CD3e depleted BM from congenic CD45.1^+^ B6 mice replenished with no T cells, 2.5 x 10^3^ or 10^6^ T cells on Day +0. On days +14-16, IC was administered and allogeneic HSCT recipients were sacrificed at Day +21 for flow cytometric analysis of B cells, **(F)** NK cells, **(G)** CD8^+^ T cells and **(H)** CD4^+^ regulatory T cells in the spleen. Results pooled from 2 similar experiments, 5-10 mice/group. NS, not significant *p < 0.05, ***p < 0.001.

Because IC have never been used after allogeneic HSCT, we established a MHC-mismatched haploidentical allogeneic HSCT model (H-2^b^ ➔ H-2^b^ x H-2^a^) whereby lethally irradiated B6AJF1 recipients were transplanted with T cell depleted B6 BM and 0 – 2.5 x 10^6^ B6 T cells on day +0 (**Figure 1A**). Because the donor and host cells are MHC-mismatched (in the GVH direction), the presence of T cells in the BM graft leads to weight loss (**Figure 1B**) and lethal GVHD in less than 30 days (**Figure 1C**). The addition of IC following such a transplant is safe in the absence of T cells (**Figure 1C**), but in the presence of 2.5 x 10^6^ T cells there is still GVHD lethality (**Figures 1C, D**). Decreasing the amount of T cells in the donor graft reduces GVHD lethality (**Figure 1D**), suggesting there is a T cell threshold where one could safely administer IC. In fact, 2.5 x10^2^ and 2.5 x 10^3^ T cells are well tolerated with IC and do not induce lethal GVHD (**Figure 1D**). Analysis of immune reconstitution shows a marked decrease in B220^+^ B cells in allogeneic HSCT recipients of 2.5 x 10^6^ T cells (**Figure 1E**), a surrogate of GVHD in other murine allogeneic HSCT models (32, 33), as well as marked decreases in NK cells (**Figure 1F**) and CD8^+^ T cells (**Figure 1G**), the cells that would typically respond to IC bound to tumor (16, 25). Both T cell depleted grafts and T cell replete grafts generate a low percentage of regulatory T cells (**Figure 1H**).

**Figure 2:**
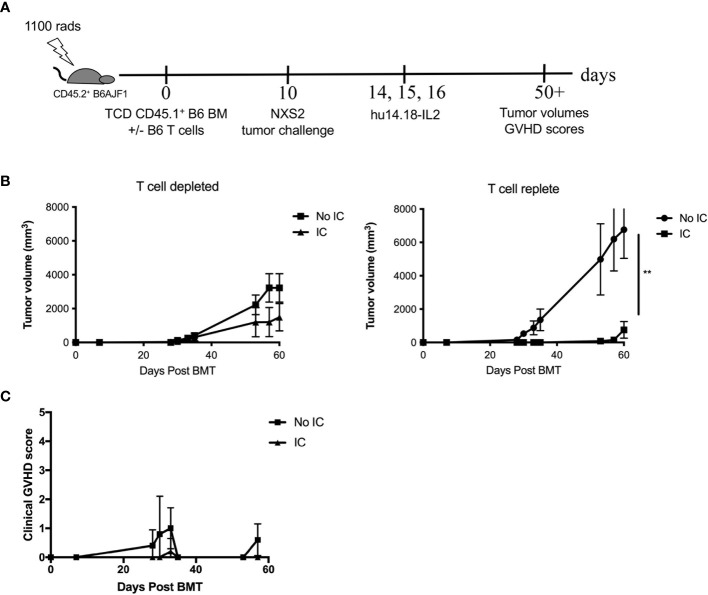
GD2^+^ NXS2 neuroblastoma growth after allogeneic HSCT and hu14.18-IL2. **(A)** Lethally irradiated CD45.2^+^ B6AJF1 mice (H-2^b x a^) were transplanted with either CD3e cell depleted (T cell depleted) or CD3e depleted BM replenished with 2.5 x 10^3^ T cells (T cell replete) from congenic CD45.1^+^ B6 mice (H-2^b^) on Day +0. On Day +10, 2 x 10^6^ NXS2 tumor cells were inoculated into the right flank. On days +14-16, PBS (No IC) or hu14.18-IL-2 (IC) was administered and **(B)** tumor growth was monitored by using a digital caliper as well as **(C)** clinical GVHD scores. N=5 mice/group. ** p = 0.01.

While human neuroblastomas ubiquitously express GD2, murine neuroblastomas show variable expression of GD2 (**Figure S1A**). NXS2 was selected so that allogeneic donor cells could be used from a C57BL/6 background, allowing for potential usage of knockout mice as NK donors in future experiments. Because children with solid tumors have the best outcomes when transplanted in remission, GD2^+^ NXS2 inoculation was performed on Day +10 after HSCT to mimic tumor relapse post-HSCT. Initially we compared syngeneic and allogeneic HSCT and found that both groups developed tumors, but NXS2 tumors in allogeneic HSCT recipients were smaller (**Figure S1B**), supporting rationale for a GVT effect in this model. We next examined allogeneic HSCT recipients (with add back of a nonlethal dose of T cells in the graft to avoid lethal GVHD but maintain some residual dose to mimic clinical T cell depletion). We performed NXS2 inoculation on Day +10, followed by 3 doses of IC on Days +14-16 (**Figure 2A**) to provide anti-GD2 tumor targeting for donor cells from the graft. Without IC, NXS2 tumors became large (**Figure 2B**). Administering IC significantly enhances the GVT effect by reducing tumor growth after T cell replete allogeneic HSCT, but small tumors still develop (**Figure 2B**). No differences were seen after T cell depleted allogeneic HSCT (**Figure 2B**), suggesting both donor T and NK cells are needed for optimal GVT effects of the IC. Importantly the IC mediates GVT without exacerbating GVHD (**Figure 2C**) after T cell replete allogeneic HSCT. *Ex vivo* activation of additional effector cells (e.g. donor-derived NK cells) that can recognize the tumor as allogeneic and/or respond to the IC *via* ADCC may enhance the GVT effect and potentially prevent tumor growth entirely.

**Figure 3:**
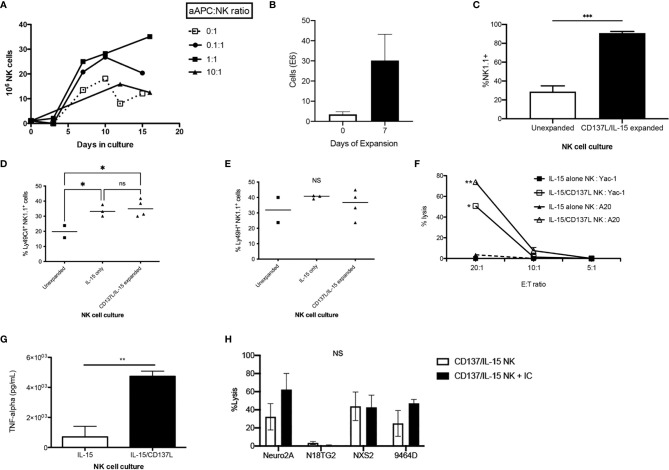
*In vitro* characterization of *ex vivo* CD137L/IL-15/IL-15Rα expanded NK cells. Donor B6 NK cells were expanded *in vitro* with IL-15/IL-15Rα alone (0:1) or with IL-15/IL-15Rα plus an aAPC expressing 4-1BBL (CD137L) at a logarithmically increasing dose of aAPC : NK cell ratios (0.1:1-10:1). **(A)** Cell counts were enumerated twice per week. Results pooled from 4 separate cultures. **(B)** After 1 week, NK cells expanded without aAPC or IL-15/IL-15Rα (unexpanded) were compared to NK cells expanded with the aAPC at a 1:1 ratio (CD137L/IL-15/IL-15Rα expanded) for **(C)** NK1.1 purity, **(D)** inhibitory Ly49C/I expression and **(E)** activating Ly49H expression. Results pooled from 2 separate experiments, 2-7 mice/group. **(F)** Balb/c NK cells (H-2^d^) expanded with IL-15/IL-15Rα or with IL-15/IL-15Rα and CD137L aAPCs at a 1:1 NK:aAPC ratio for 1 week were compared for their ability to lyse syngeneic A20 (H-2^d^) or allogeneic Yac-1 (H-2^a^) lymphoma cells at various E:T ratios using a 4 hour LDH release cytotoxicity assay, performed in triplicate. **(G)** NK cells expanded without aAPC (IL-15) were compared to NK cells expanded with the aAPC at a 1:1 ratio (IL-15/CD137L) and examined for TNF-α production by ELISA, performed in triplicate. **(H)** B6 (H-2^b^) NK cells expanded with IL-15/IL-15Rα and CD137L aAPCs at a 1:1 NK:aAPC ratio for 1 week were tested for their ability to lyse murine neuroblastoma cell lines Neuro2a, N18TG2, NXS2, and 9464D (H-2^a^) using a 4 hour calcein-AM release cytotoxicity assay, performed in triplicate. *p < 0.05, **p < 0.01, ***p < 0.001, NS, not significant.

Human aAPCs that express the co-stimulatory molecule 4-1BB ligand (CD137L) have been shown to potently expand and activate human NK cells (34–37), however has not been explored on murine NK cells. Using a murine aAPC transfected with CD137L in the presence of IL-15/IL-15Rα expands purified murine NK cells *ex vivo* (**Figure 3A**), with the highest yields after 1 week at a 1:1 ratio of NK:aAPC (**Figure 3B**). Activating NK cells with the CD137L^+^ aAPC without IL-15/IL-15Rα is insufficient to sustain NK cell growth (data not shown). The purity of NK cells after 7 days of *ex vivo* expansion is 90% (**Figure 3C**). While the percentage and absolute numbers of NK cells increase after *ex vivo* expansion, the percentage of NK cell subsets within that expanded population also changes. There is a mild but statistically significant increase in the percentage of Ly49C+I^+^ NK cells after *ex vivo* activation with IL-15/IL-15Rα alone or with CD137L/IL-15/IL-15Rα compared to unexpanded NK cells (**Figure 3D**). In contrast, we did not see any differences in the percentage of Ly49H^+^ NK cells after *ex vivo* activation (**Figure 3E**). *Ex vivo* activation also occurs, as evidenced by enhanced NK cytotoxicity as measured by potency assays *in vitro* (**Figure 3F**) and *in vivo* (**Figure S2**), and augmented TNF-α production (**Figure 3G**). There are no significant changes in the percentage of cytotoxic (TRAIL^+^, FasL^+^ or CD107a^+^) or TNF-α producing Ly49 NK subsets (**Figure S3**). Interestingly, *ex vivo* activated (H-2^b^) NK cells demonstrate lysis of various syngeneic (H-2^b^: 9464D) and allogeneic murine NBL cell lines (H-2^a^: Neuro-2A, N18TG2, NXS2), however no significant improvement is seen with the addition of IC *in vitro* (**Figure 3H**). Because GVT/GVHD is a complex phenomenon that cannot be recapitulated *in vitro*, this observation led us to test if there were characteristics of the allogeneic HSCT milieu that could enhance a NK-mediated GVT effect with the addition of IC against GD2^+^ NBL *in vivo*.

**Figure 4:**
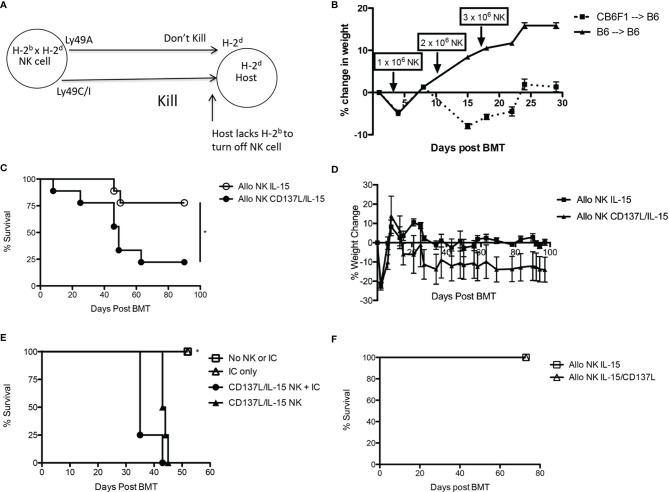
Effects of *ex vivo* expanded NK cells after syngeneic and allogeneic MHC-matched and mismatched HSCT. **(A)** Child into parent HSCT schema showing how “missing self” in host prevents engagement of Ly49 inhibitory receptors on donor NK cells. **(B)** Lethally irradiated B6 mice (H-2^b^) were transplanted with CD3e cell depleted BM from B6 (H-2^b^) or CB6F1 mice (H-2^bxd^) on Day +0. On Days +3, +10, and +17 HSCT recipients received donor-derived, CD137L/IL-15/IL-15Rα expanded NK cells in increasing dose increments of 1, 2 or 3 x 10^6^ cells, and were followed for weight loss and survival. N=5 mice/group. **(C)** Lethally irradiated Balb/c mice (H-2^d^) were transplanted with CD3e cell depleted BM from CB6F1 mice (H-2^b x d^) on Day +0. On Day +1 HSCT recipients received 5 x 10^6^ CB6F1 NK cells (H-2bxd) cultured in IL-15 alone, or activated with CD137L/IL-15/IL-15Rα, and were followed for survival and **(D)** weight loss. Lethally irradiated Balb/c mice (H-2^d^) were transplanted with CD3e cell depleted BM from CB6F1 mice (H-2^b x d^) on Day +0. On Day +1 HSCT recipients received 5 x 10^6^ CB6F1 NK cells (H-2^bxd^) cultured in IL-15 alone, or activated with CD137L/IL-15/IL-15Rα, and were followed for survival. Results pooled from 2 separate experiments, 9 mice/group. **(E)** Lethally irradiated A/J mice (H-2^a^) were transplanted with CD3e cell depleted BM from B6 mice (H-2^b^) on Day +0. On Days +14-16, PBS (No IC) or hu14.18-IL-2 (IC) was administered. On Day +16, select groups were infused with 2.5 x 10^6^ CD137L/IL-15/IL-15Rα expanded B6 NK cells and followed for survival. N= 5 mice/group. **(F)** Lethally irradiated Balb/c mice (H-2^d^) were transplanted with CD3e cell depleted BM from DBA mice (H-2^d^) on Day +0. On Day +1 HSCT recipients received 5 x 10^6^ CB6F1 NK cells (H-2^b x d^) cultured in IL-15/IL-15Rα alone, or activated with CD137L/IL-15/IL-15Rα, and were followed for weight loss and survival. N=5 mice/group. *p < 0.05.

**Figure 5:**
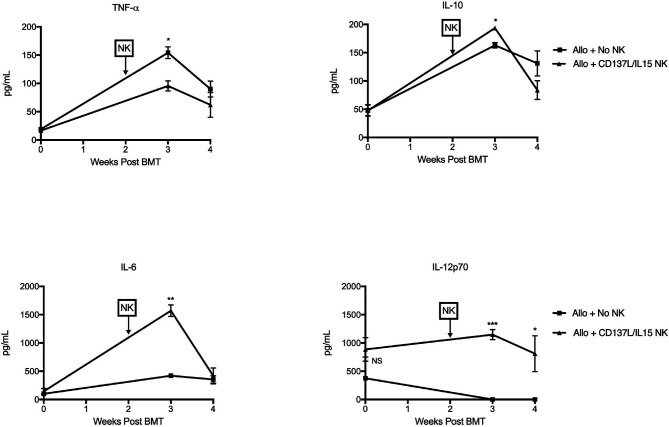
*Ex vivo* CD137L/IL-15/IL-15Rα expanded NK cells mediate cytokine release syndrome *in vivo*. Lethally irradiated B6AJF1 mice (H-2^b x a^) were transplanted with CD3e cell depleted BM from B6 mice (H-2^b^) on Day +0. On Day +10, 2 x 10^6^ NXS2 tumor cells were inoculated into the right flank. On days +14-16, hu14.18-IL-2 (IC) was administered. On Day +16, groups were infused with PBS or 2.5 x 10^6^ CD137L/IL-15/IL-15Rα expanded B6 NK cells. Peripheral blood was collected pre-alloHSCT, and then 3 and 4 weeks post-alloHSCT. Serum was isolated from each blood sample and all timepoints were frozen, batched and analyzed in duplicate by multiplex cytokine array. N= 5 mice/group. *p < 0.05, **p < 0.01, ***p < 0.001.

During allogeneic HSCT, the GVT effect is mediated by T cells and NK cells while GVHD is mainly mediated by α/β^+^ T cells. To determine the contribution of NK alloreactivity to a GVT effect without contribution of donor T cells, we designed a F1 into parent allogeneic HSCT model so that (1) any residual donor T cells in the BM graft would be tolerized to host MHC and minor histocompatibility antigens in the thymus and thus not mediate GVHD (38), and (2) donor NK cells could still mediate alloreactivity since the host would lack cognate MHC ligands needed to engage donor Ly49 inhibitory receptors (39) (**Figure 4A**). When we infused F1 NK cells into one parent strain (H-2^bxd^ ➔ H-2^b^), we observed that allogeneic *ex vivo* activated NK cells could mediate a mild weight loss (**Figure 4B**), but no lethality was observed (data not shown). Lethality was observed after infusion of F1 NK cells into the other parent strain (H-2^bxd^ ➔ H-2^d^), with significantly more lethality observed with NK cells activated with CD137/IL-15/IL-15Rα after allogeneic HSCT than infusing NK cells activated with IL-15/IL15Rα alone (**Figure 4C**), indicating the contribution of CD137L during NK expansion and host MHC molecules in driving toxicity. More weight loss was seen with CD137/IL-15/IL-15Rα NK cells (**Figure 4D**). The infusion of *ex vivo* activated NK cells in a fully MHC-mismatched, T cell depleted, allogeneic HSCT model (H-2^b^ ➔ H-2^a^) leads to lethality with or without hu14.18-IL2 (**Figure 4E**), suggesting IC does not contribute to lethality. Interestingly, when the allogeneic HSCT donor and recipients were MHC-matched, minor histocompatibility antigen-mismatched, no differences in weight loss (data not shown) or lethality were observed (**Figure 4F**). Histopathologic examination of classic acute GVHD target tissues (liver, gut, skin) did not show any lymphocytic infiltrate (data not shown), suggesting there was no direct attack of host tissues. Analysis of serum cytokines, however, did show cytokine release syndrome (CRS) with statistically significant increases in IL-6, IL-10 and IL-12p70 and a decrease in TNFα noted 1 and/or 2 weeks after *ex vivo* activated NK infusion as compared to recipients of allogeneic HSCT alone (**Figure 5**). No differences in IFNγ, IL-1β, IL-2, IL-4, IL-5 and CXCL1 were observed (**Figure S4**).

**Figure 6:**
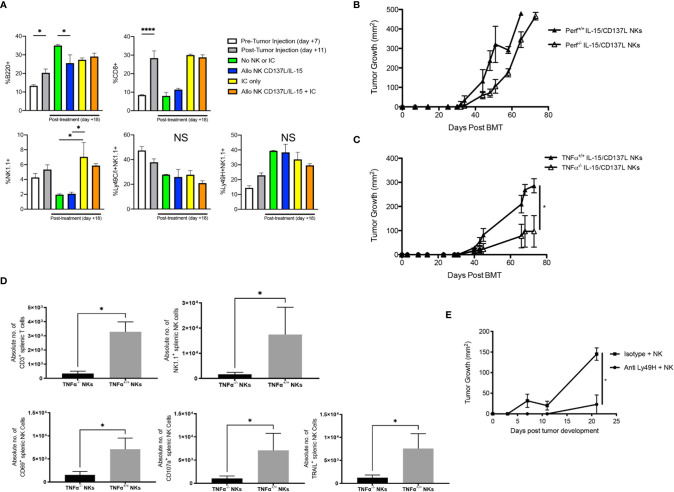
Impact of infusing *ex vivo* CD137L/IL-15/IL-15Rα expanded NK cells on GD2^+^ NXS2 neuroblastoma growth after allogeneic HSCT. Lethally irradiated A/J mice (H-2^a^) were transplanted with CD3e cell depleted B6 BM (H-2^b^) with 2.5 x 10^2^ B6 T cells on Day +0. On Day +10, 2 x 10^6^ NXS2 tumor cells were inoculated into the right flank. On Days +14-16, hu14.18-IL-2 (IC) was administered. **(A)** Flow cytometric analysis of the spleen was performed on Day +7 (pre-NXS2), +11 (post-NXS2, pre-IC or NK cells) or +18 (post-IC and/or NK cells). **(B)** On Day +16, select groups were infused with 2.5 x 10^6^ CD137L/IL-15/IL-15Rα expanded NK cells from B6 wild type (Perf^+/+^) or B6 Perforin (Perf^-/-^) donors, or **(C)** B6 wild type (TNFα^+/+^) or B6 TNFα^-/-^ donors. **(D)** Both spleens and tumors were harvested from recipients of B6 wild type (TNFα^+/+^) or B6 TNFα^-/-^ donors at Day +30 and analyzed for T cells and NK cell subsets. **(E)** Mice were transplanted and challenged with NXS2 as above but on Day +16 were infused with 2.5 x 10^6^ CD137L/IL-15/IL-15Rα expanded NK cells from B6 wild type and then treated with anti-Ly49H depletion or an isotype control. All mice were followed for NXS2 tumor growth. N=3-5 mice/group. *p < 0.05. ****p < 0.001. NS, not significant.

Immune profiling of allogeneic HSCT recipients showed mild increases in B cells and CD8^+^ T cells after tumor inoculation, but no changes in NK cells (**Figure 6A**). While adoptive transfer of wild type NK cells did not increase the total percentage of NK cells in the host, total NK cells did increase after IC administration but without enrichment of inhibitory Ly49C/I^+^ or activating Ly49H^+^ NK subsets (**Figure 6A**). Because *ex vivo* activated NK cells showed superior cytotoxicity *in vitro* (**Figure 3F**) and *in vivo* (**Figure S2**), as well as high levels of TNFα production *ex vivo* (**Figure 3G**), we wanted to determine if the GVT effect was mediated by contact-dependent killing (*via* perforin) or contact-independent cytokine release (*via* TNF-α release), and whether abrogating these pathways would impact GVT. Infusion of Perforin^-/-^ *ex vivo* activated NK cells with IC did lead to a slight delay in tumor growth, but ultimately tumors overtook the mice (**Figure 6B**). But when we infused *ex vivo* activated TNFα^-/-^ NK cells after allogeneic HSCT with IC, we observed improved tumor control compared to *ex vivo* activated TNFα^+/+^ NK cells (**Figure 6C**), suggesting TNFα may be contributing to CRS in a manner that attenuates the GVT potential of the infused NK cells. Flow cytometric analysis of splenocytes of mice treated with *ex vivo* activated TNFα^-/-^ NK cells showed a decrease in T cells and NK cells, with specifically less CD69^+^, CD107a^+^, and TRAIL^+^ NK cells seen in the periphery (**Figure 6D**). However, there were no differences between these NK subsets within the tumor (**Figure S5**). Depletion of Ly49H^+^ NK cells, which represent an NK subset bearing an activation receptor that can engage MHC (H-2^b^) on B6AJF1 host tissues, after NK infusion also led to improved tumor control early after tumor development (**Figure 6E**), suggesting blockade of TNFα-producing or depletion of activated NK cell subsets may help regulate toxicity while preserving GVT responses against NBL.

## Discussion

While haploidentical allogeneic HSCT is effective against leukemia (40), despite the publication of preclinical data (10, 11) and clinical data from case series describing the impact of allogeneic HSCT on NBL (12, 13), significant barriers are preventing allogeneic HSCT from more widespread testing as potential therapy for children with high risk or metastatic NBL. Barriers include the absence of conclusive evidence of a GVT effect against NBL and the development of GVHD that contributes to treatment-related mortality (41). We hypothesized that these barriers may be overcome by: 1) using T cell-depleted haploidentical allogeneic HSCT to enhance GVT and minimize GVHD; 2) focusing the localization and activity of the GVT inducing cells in the allogeneic HSCT *via* co-administration of the anti-GD2 IC hu14.18-IL2; 3) augmenting the capability of the GVT causing cells by selecting donors with the appropriate haploidentical relationship to the patient to enable NK allo-recognition in the GVT direction; and 4) co-infusing *ex-vivo* activated NK cells.

We show for the first time that usage of an IC, in this case hu14.18-IL2, is feasible and effective after allogeneic HSCT; IC induces GVT without GVHD as long as the T cell dose is minimized in the donor bone marrow graft. Because IL-2 could activate alloreactive T cells and exacerbate GVHD, but also expand regulatory T cells and abrogate GVHD, it was not clear what the effect of infusing IC would be after allogeneic HSCT. With higher T cells doses, the IL-2 present on the IC may have unintentionally stimulated alloreactive T cells from the donor, leading to GVHD. Also, poorer immune reconstitution was observed, which could reflect immunosuppression from GVHD or reduced spleen size as allogeneic HSCT recipients were dying from GVHD. With lower T cell doses, while anti-tumor activity against GD2^+^ NBL was observed as compared to allogeneic HSCT recipients without IC, tumors still developed. Because the mechanism of action of IC involves ADCC by NK cells, allogeneic HSCT recipients are lymphopenic, and the post-allogeneic HSCT milieu has high levels of IL-15 (21, 42), we hypothesized that infusions of *ex vivo* activated NK cells from the donor could enhance the GVT effect of the IC. Instead, we observed that adoptive transfer of *ex vivo* activated NK cells led to lethality in the presence or absence of IC.

NK cells have been adoptively transferred to recipients of allogeneic HSCT in preclinical models, with promising anti-tumor activity observed (43–46). Adoptive transfer of NK cells can also inhibit acute GVHD by limiting expansion and infiltration of donor T cells (47–49), producing TGF-β (45), controlling infections (50), depleting recipient dendritic cells (39), and improving lymphopenia (51). One limitation of applying these murine studies to our data is that all but two of these studies infused inactivated NK cells, and none of those studies used a co-stimulatory molecule like CD137L to activate the NK cells. Using F1 into parent HSCT models, where T cells cannot cause GVHD or alloreactivity, we observed weight loss or lethality depending on the recipient strain, suggesting *ex vivo* activated NK cells can mediate toxicity independent of T cell allorecognition. While this has not been observed in prior preclinical studies of adoptively transferred NK cells, it is possible that the biology of NK cells is different *in vivo* after activation by *ex vivo* as compared to inactivated NK cells. *In vitro*, *ex vivo* activated human NK cells can overcome KIR-mediated inhibitory signals (52). In clinical studies, infusion of NK cells expanded with either IL-2 (53, 54), or IL-15 and IL-21 after HLA-mismatched allogeneic HSCT (55–57) induced low rates and/or grades of GVHD, whereas infusion of NK cells activated with CD137L/IL-15 after HLA-matched allogeneic HSCT led to higher rates and grades of GVHD (58). The exact mechanism of NK-mediated GVHD is unclear but our data suggests it could have been in part driven by CRS.

Because we used T cell depleted bone marrow and did not detect NK cells in host tissues, we hypothesized that *ex vivo* activated NK cells mediated CRS that inhibited GVT. In fact, elevated levels of IL-6, IL-10 and IL-12p70 and decreased levels of TNF-α were observed in allogeneic HSCT recipients who received *ex vivo* activated NK cells than in uninfused allogeneic HSCT recipients. We did observe a minor population of regulatory T cells after IC administration that was not influenced by the number of T cells in the donor graft. Given the high IL-10 production observed after NK infusion, future studies should examine the contribution of IC in activating regulatory T cells and their role in GVT/GVHD/CRS in this model. In addition, increased TNF-α production by NK cells has been previously observed after haploidentical allogeneic HSCT (59), yet the decreased level noted in our model was still clinically significant. To determine if CRS was attenuating the GVT effect, adoptive transfer of purified, *ex vivo* activated TNFα ^-/-^ NK cells was performed and significantly attenuated tumor growth, suggesting that TNF-α production from *ex vivo* CD137L/IL-15/IL-15Rα activated NK cells may be contributing to a CRS that hinders anti-tumor effects. One potential mechanism could have been disruption of TNF-α mediated priming of regulatory T cells through TNFR2 (60, 61), reducing tolerance by/to the tumor. Future studies should examine if TNF-α may be activating regulatory T cells which in turn suppress elimination of NBL by T and NK cells. In addition, depleting *ex vivo* activated NK cells that express the activating receptor Ly49H after infusion improves early anti-tumor responses, overall suggesting that hyperactivated NK cell subsets may have to be carefully monitored after allogeneic HSCT as they may contribute to toxicities like CRS than can undo GVT effects. To our knowledge, this is the first example of CRS using adoptive transfer of *ex vivo* activated NK cells in the allogeneic HSCT setting. Because of the clinical availability of TNF-α inhibitors like infliximab, or soluble TNF-α receptor like etanercept, TNF-α blockade could be explored clinically to improve the GVT potential of *ex vivo* activated NK cells, but additional agents would likely need to be explored to better control CRS, like tocilizumab.

The preclinical data shown here provide preliminary groundwork for more mechanistic studies to enable clinical translation and evolution of existing pediatric trials using T cell depleted (e.g. α/β T cell depletion) haploidentical allogeneic HSCT for NBL by demonstrating the safety and efficacy of the combination of IC and *ex vivo* activated NK cell infusions to induce GVT effects against NBL. Clinical trials incorporating α/β T cell depletion haploidentical HSCT are underway at several pediatric centers as a means of depleting GVHD-causing α/β T cells while enriching the donor graft with GVT-promoting γ/δ T cells and NK cells (62), including for children with NBL (63, 64) (NCT02508038). A pilot trial testing the combination of IC and *ex vivo* activated haploidentical NK cell infusions in non-transplanted NBL patients is also underway (NCT03209869) (65). Further studies are warranted with these clinically available therapy platforms given the poor prognosis for high risk NBL and lack of effective salvage regimens.

## Data Availability Statement

The raw data supporting the conclusions of this article will be made available by the authors, without undue reservation.

## Ethics Statement

The animal study was reviewed and approved by University of Wisconsin-Madison IACUC.

## Author Contributions

PB, SR, JH, and MC performed research, collected, analyzed, and interpreted data, and revised the manuscript. AR performed research and revised the manuscript. RO developed and provided the aAPC and revised the manuscript. MB, TF, SG, and PS analyzed and interpreted data and revised the manuscript. CC designed and supervised research; analyzed and interpreted data, drafted and revised the manuscript. All authors contributed to the article and approved the submitted version.

## Funding

This work was supported by grants from the NIGMS/NIH T32 GM008692 and NCI/NIH T32 CA009135 (MC), NCI/NIH T32 CA009614 (SR), St. Baldrick’s – Stand up to Cancer Pediatric Dream Team Translational Research Grant SU2C-AACR-DT-27-17 (AR, TF, PS, CC), the Intramural Research Program at the NIH (TF), NCI/NIH R35 CA197078 (PS), the NCI/NIH R01 CA215461, St. Baldrick’s Foundation, American Cancer Society Research Scholar grant RSG-18-104-01-LIB, Hyundai Hope on Wheels and the MACC Fund (CC). We would like to thank the UWCCC flow cytometry core laboratory, small molecule screening facility and experimental animal pathology core laboratory, who are supported in part through NCI/NIH P30 CA014520. The St. Baldrick’s Foundation collaborates with Stand Up To Cancer.

## Conflict of Interest

SG is an employee of Provenance Biopharmaceuticals, and has a patent related to hu14.18-IL2. CC reports honorarium from Nektar Therapeutics and Novartis. These companies had no input in the study design, analysis, manuscript preparation or decision to submit for publication.

The remaining authors declare that the research was conducted in the absence of any commercial or financial relationships that could be construed as a potential conflict of interest.

## Publisher’s Note

All claims expressed in this article are solely those of the authors and do not necessarily represent those of their affiliated organizations, or those of the publisher, the editors and the reviewers. Any product that may be evaluated in this article, or claim that may be made by its manufacturer, is not guaranteed or endorsed by the publisher.

## Supplementary Material

Supplementary Figure 1 Comparison of GD2 expression on murine NBL and syngeneic versus allogeneic HSCT on NBL tumor growth. **(A)** Flow cytometric expression of GD2 on murine NBL cell lines. **(B)** A/J mice were lethally irradiated and transplanted with 5 x106 B6 BM cells and 2.5 x103 B6 T cells (allogeneic BMT) or 5 x10^6^ A/J BM cells and 2.5 x10^3^ A/J T cells (syngeneic BMT) on Day +0. On Day +10, mice were inoculated subcutaneously with 2 x 10^6^ NXS neuroblastoma cells. Mice were followed for tumor growth. N = 5 mice/group. *p < 0.05.

Supplementary Figure 2 *In vivo* potency of *ex vivo* activated NK cells. Balb/c mice were lethally irradiated and transplanted with 5 x 10^6^ CB6F1 BM cells (allogeneic HSCT) on Day +0 as well as 2.5 x 10^6^ A20 lymphoma cells on Day +5. Then PBS (No NKs) or 6 x10^6^ CB6F1 IL15/IL15Rα activated or CD137L/IL15/IL15Rα activated NK cells were given on Day +13. All mice were followed for survival. N=5 mice/group. *p < 0.05 as compared to No NKs group.

Supplementary Figure 3 Ly49 NK subset analysis. **(A)** Sample gating strategy for analyzing Ly49C/I+NK1.1+ cells pre- and post-expansion by CD137L/IL15/IL15Rα aAPCs at a 1:1 ratio for 1 week. **(B)** Flow cytometric analysis of Ly49H- and Ly49H+ NK cell subsets for cytotoxicity markers (TRAIL, FasL and CD107a) and cytokine production (TNFα) from a single expansion.

Supplementary Figure 4 Cytokine production pre- and post-adoptive transfer of *ex vivo* activated NK cells. B6AJF1 mice were lethally irradiated on Day +0 and transplanted with 5 x10^6^ B6 BM cells and 1 x10^6^ B6 T cells on Day +0. Serum was collected as a baseline cytokine measurement. On Day +10, mice were inoculated with 2 x10^6^ NXS2 neuroblastoma cells. On Day +14, mice wereinfused with PBS or 2.5 x 10^6^ B6 CD137L/IL15/IL15Rα activated NK cells. Serum was collected 1 (Day +21) and 2 weeks (Day +28) after infusion. Serum was batched and run in duplicate per timepoint for each group. N=5 mice/group. *p < 0.05, otherwise no significant differenceswere seen.

Supplementary Figure 5 Comparison of tumor infiltrating wild type and TNFα-deficient NK cells after allogeneic HSCT and IC treatment for NBL. Lethally irradiated A/J mice (H-2a) were transplanted with CD3e cell depleted B6 BM (H-2b) with 2.5 x 10^6^ B6 T cells on Day +0. On Day +10, 2 x 10^6^ NXS2 tumor cells were inoculated into the right flank. On Days +14-16, hu14.18-IL-2 (IC) was administered. On Day +16, select groups were infused with 2.5 x 10^6^ CD137L/IL-15/IL-15Rα expanded NK cells from B6 wild type (TNFα+/+) or B6 TNFα-/- donors. Both spleens and tumors were harvested at Day +30 and analyzed for T cells and NK cell subsets. N=3-5 mice/group. No significant differences were seen.
