# Supplementary figures and images for: Combining Immunocytokine and Ex Vivo Activated NK Cells as a Platform for Enhancing Graft-Versus-Tumor Effects Against GD2+ Murine Neuroblastoma
Source: Front Immunol. 2021 Aug 19;12:668307. doi: 10.3389/fimmu.2021.668307 (PMC8417312; doi:10.3389/fimmu.2021.668307)

A

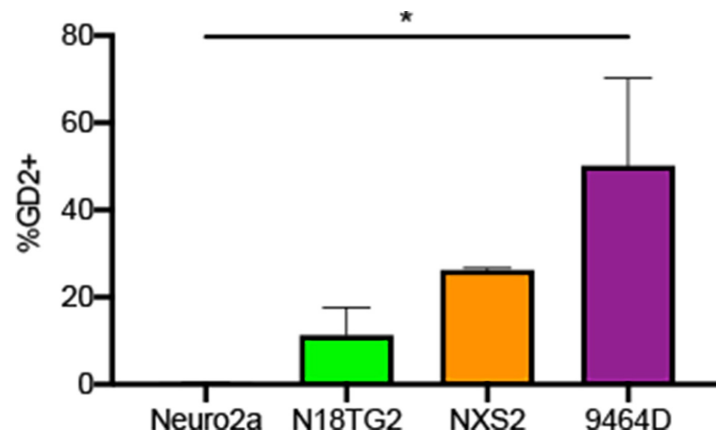

B

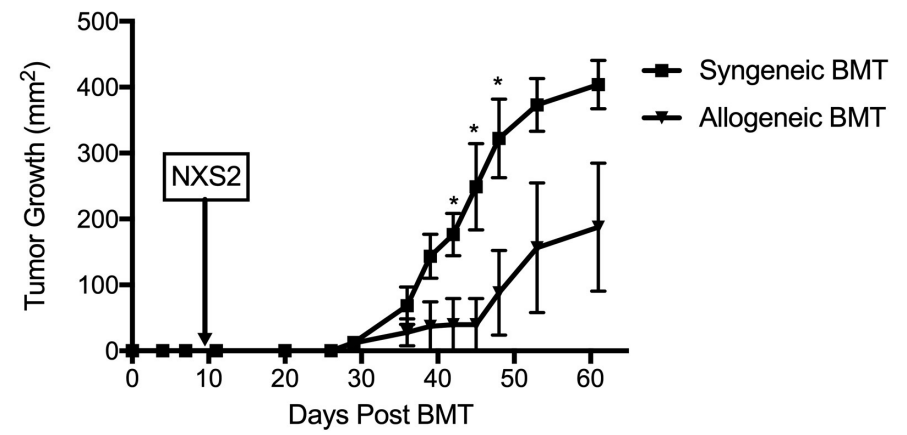

Figure S1

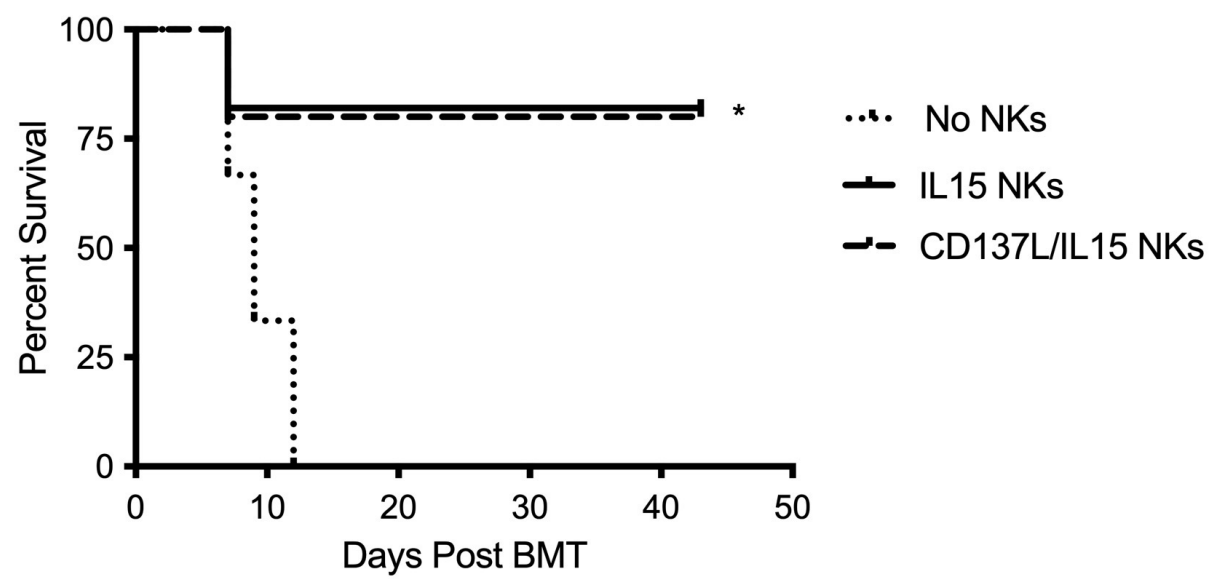

Figure S2

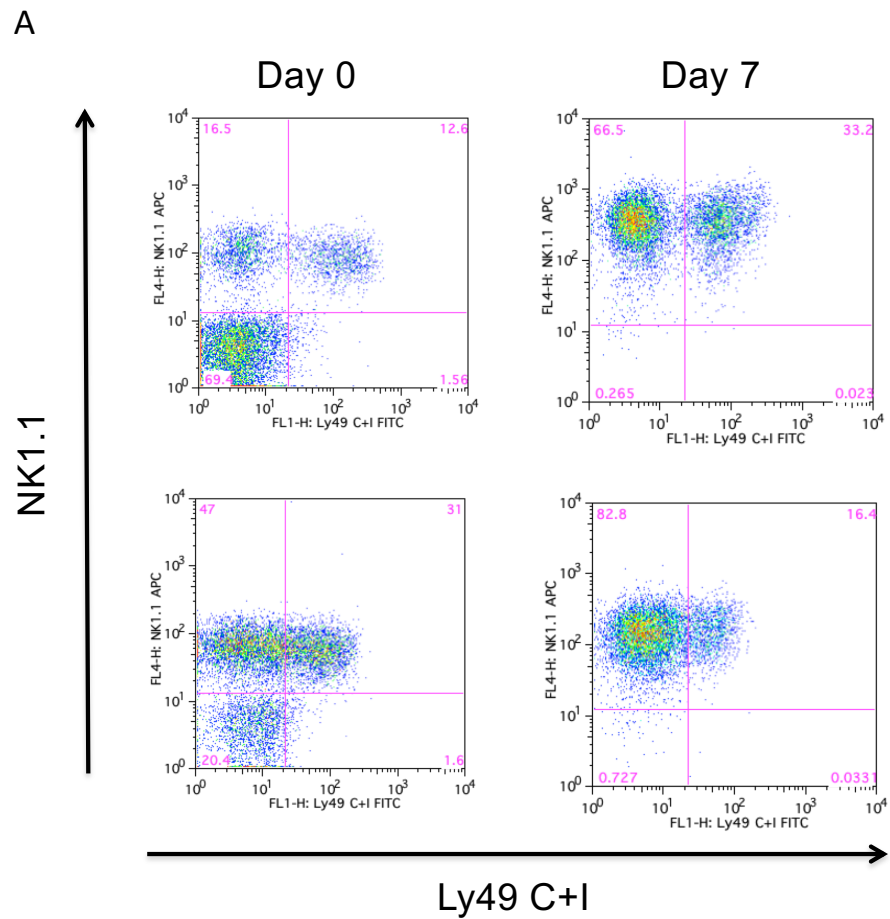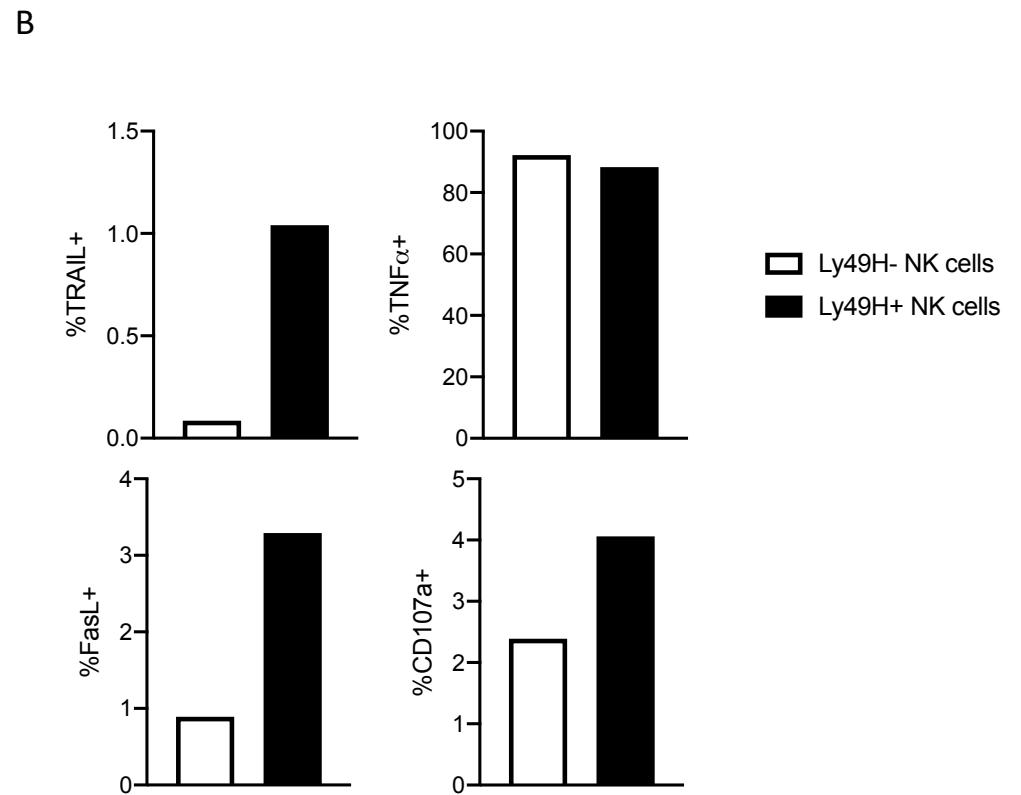

Figure S3

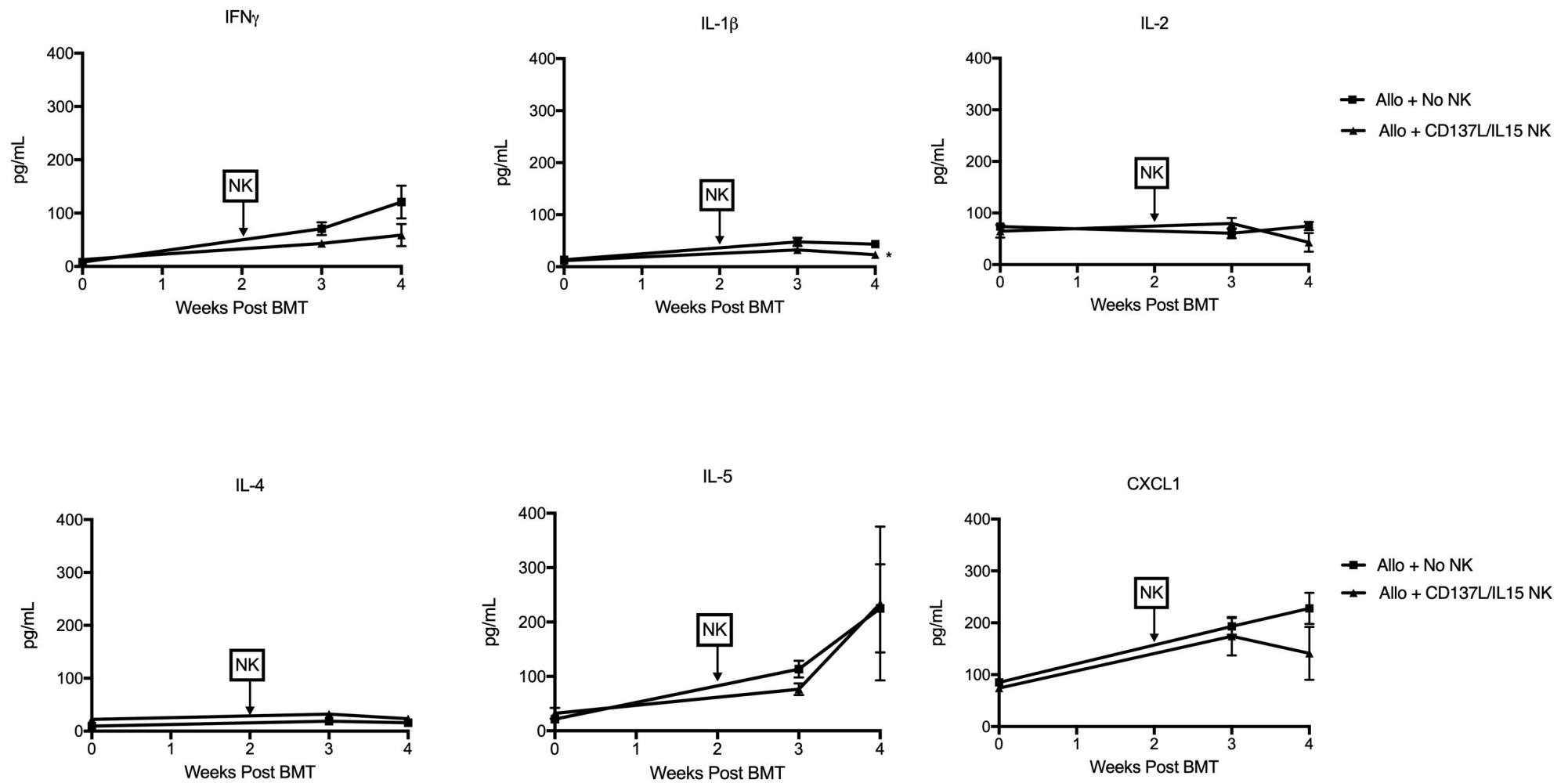

Figure S4

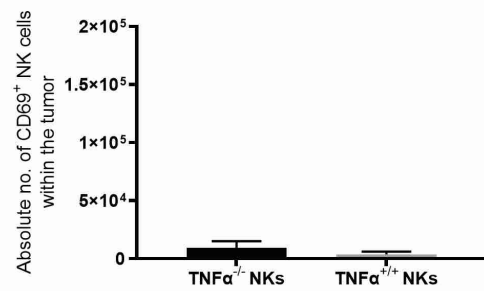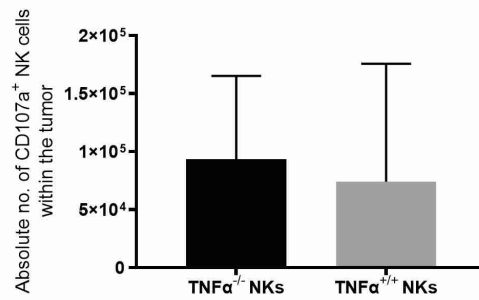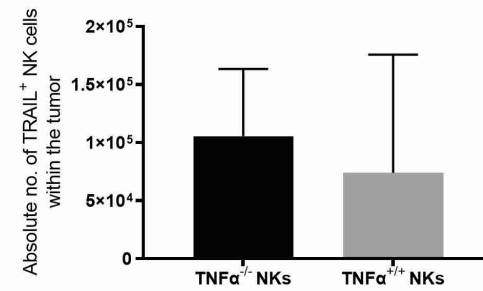

Figure S5

Supplement: Supplementary Figure 1 — Comparison of GD2 expression on murine NBL and syngeneic versus allogeneic HSCT on NBL tumor growth. (A) Flow cytometric expression of GD2 on murine NBL cell lines. (B) A/J mice were lethally irradiated and transplanted with 5 x10^6^ B6 BM cells and 2.5 x10^3^ B6 T cells (allogeneic BMT) or 5 x10^6^ A/J BM cells and 2.5 x10^3^ A/J T cells (syngeneic BMT) on Day +0. On Day +10, mice were inoculated subcutaneously with 2 x 10^6^ NXS neuroblastoma cells. Mice were followed for tumor growth. N = 5 mice/group. *p < 0.05. [file DataSheet_1.pdf]
